# Toward Customized Care: Comment on "(Re) Making the Procrustean Bed? Standardization and Customization as Competing Logics in Healthcare"

**DOI:** 10.15171/ijhpm.2017.84

**Published:** 2017-07-15

**Authors:** Etienne Minvielle

**Affiliations:** Ecole des hautes études en santé publique (EHESP), Institut Gustave Roussy, Villejuif, France.

**Keywords:** Customization, Personalization, Care, Healthcare Delivery

## Abstract

Patients want their personal needs to be taken into account. Accordingly, the management of care has long involved some degree of personalization. In recent times, patients’ wishes have become more pressing in a moving context. As the population ages, the number of patients requiring sophisticated combinations of longterm care is rising. Moreover, we are witnessing previously unvoiced demands, preferences and expectations (eg, demand for information about treatment, for care complying with religious practices, or for choice of appointment dates). In view of the escalating costs and the concerns about quality of care, the time has now come to rethink healthcare delivery. Part of this reorganization can be related to customization: what is needed is a customized business model that is effective and sustainable. Such business model exists in different service sectors, the customization being defined as the development of tailored services to meet consumers’ diverse and changing needs at near mass production prices. Therefore, its application to the healthcare sector needs to be seriously considered.

## Introduction

Imagine a healthcare delivery system designs to be customized for each *individual* patient, instead of ‘one size fits all.’ The editorial of Russell Mannion and Mark Exworthy^1^ suggests that we need to think more about how to design our delivery systems to fit the needs and expectations of patients. In this commentary, I extend their point and argue that although it may be obvious that one size does not fit all in patient care, intentional efforts to customize care further by tailoring it to the relevant characteristics of individual patients and engaging patients more extensively in decisions about their own care could result in a number of positive payoffs.

In the business world, the logic of “mass customization” has been used to guide companies’ efforts to connect their products more directly to specific customer wants and needs by allowing customers to choose certain attributes of the products being offered. Auto manufacturers, for example, encourage potential customers to choose such attributes as color, engine size, interior finish, and communication capabilities on line, allowing them to “customize” their purchase. The customer receives a pair of shoes that “fits,” presumably better than a pair bought off the shelf. The emergence of increasingly sophisticated IT capabilities, customization is also becoming widely used in many service industries such as financial planning, legal services, and education, with the goal of offering equal or better quality at lower cost. In the worlds of both products and services, mass customization^2^ seeks to empower frontline staff and their managers to develop tailored services to meet the diverse and changing needs of their customers at near mass-production prices.

In healthcare, the logic of mass customization is being applied in distinct, but related, ways as mentioned by Russell Mannion and Mark Exworthy.

## Developing Customized Care: An Organizational Approach

Efforts to develop customized care must be designed around a deep understanding of what happens at the ground level along the patient pathway and must incorporate patient preferences by focusing on such things as shared decision-making, definition of appointments, delays and self-management, all of which are elements of an organizational approach. Soliciting active feedback also can help to promote necessary adjustments in the organization of work.^3^ Such efforts foster the ability of patients to incorporate their own priorities while minimizing complexity and the burden of choice, arguably leading to both quality improvement and cost savings. However, all these efforts must be integrated in a delivery system.

No one would dispute that the delivery of patient care is complex. But the level of complexity is increasing at a greater rate than the managerial capacity to deal effectively with it. Among the drivers of increasing complexity are intensification of economic pressure, the need to find better ways of managing chronic conditions in aging populations, and efforts to build patient preferences and goals into the delivery process.

In the last 10 years, hospitals in industrialized nations around the globe, paid on the basis of some diagnosis-related group (DRG) or DRG-like system, have been incentivized to increase their volume of activity, or the number of patients treated per unit time.^4^

For better or for worse, its impact on patient care has been huge. To be financially successful, hospitals have to execute faster on all facets of patient care, often creating tension between clinical and administrative goals, and ultimately resulting in lower average lengths of stay for patients. In fact, the average length of stay has fallen in the last ten years in virtually every the Organization for Economic Co-operation and Development (OECD) member country, dropping from 9.2 days in 2000 to 7.3 in 2013.^5^

In the wake of increasing dissatisfaction with DRG-type payment systems, and the ongoing upward march of healthcare costs, alternative models such as bundled payments and accountable care arrangements have emerged. Now, the driver of complexity is not just pressure to do more in a shorter span of time, but also the need to coordinate care in smarter ways so that high-quality care is delivered at an acceptable cost.

These reasons explain the tension that customization can introduce as described by Russell Mannion and Mark Exworthy. Customized care must be applied to more activities that need to be coordinated, more “sites” for care to be taken into account, and all of this under pressure to get it done more quickly and at lower cost that need to standardize whenever possible.

In addition, this tension can be amplified by health professionals’ beliefs, values, culture. Some express reluctance to absolve responsibility for treatment decision making, and may not engage in inter-professional collaboration required to customize all aspects of healthcare.

The challenge, then, is clear. The process of improving a targeted pathway according to the logic of customization and then spreading the knowledge gained from such an effort within a delivery system can’t be accomplished quickly or easily.

Such improvements require well-planned and carefully orchestrated investments of time and energy by leaders, middle managers, trainers, and frontline staff. These investments will help to ensure three preconditions for the successful implementation of customized care.^6^

## Preconditions for Developing Customized Care

### 1. Defining Patient Profiles

To offer customized care, distinct categories of patient profiles have to be built. In the business world, product-consumption patterns typically are used as a criterion for the exploration of consumer behavior and as the basis for consumer relationship management. This practice needs to be adapted for more sophisticated use in healthcare.

In the world of healthcare, this effort relies primarily on clinical and genomic criteria that form the basis for personalized medicine, supplemented by additional data describing the patient’s socio-economic context. For instance, medical as well as social vulnerability and cognitive criteria can be applied to tailor the management of elderly patients according to their degree of vulnerability or disability. In addition, patients’ beliefs, values and behaviors should be accounted for as these have also significant impact on treatment preferences, uptake, adherence and outcomes. There is strong demand for various services based on these beliefs and values (eg, for care that complies with religious practices or cultural customs and traditions). The ability to categorize patients according to both individual characteristics and a wide range of social context information, combined with the ability to link this information to priority outcomes, would be a major step toward customized care.

### 2. Leveraging IT

IT makes large-scale customization possible by facilitating the use of large volumes of data to help build patient categories at relatively low cost and through real-time execution. Remote exchange of regular data via online portals and applications (“apps”) personalizes the provider-patient relationship. For example, in the case of patients with cancer, a portal enables nurse navigators and physicians to exchange information about treatment with oral chemotherapy, allowing side effects to be managed more effectively and doses to be adjusted more precisely.^7^ And digital sensors (eg, those in connected devices, mobile apps, etc) operate a variety of warning indicators. Any significant and sudden change in a given indicator can be transmitted to the health professional, who can then rapidly adjust the patient’s pathway. Validated algorithms can minimize potential for error in individual provider’s judgment. These examples only scratch the surface of IT applications that can enhance customization. An effective implementation requires to engage end users (patients and providers) in the design of IT applications and to assess their acceptability (emerging evidence shows limited uptake of IT-based interventions by patients, particularly the older adults with complex needs).

### 3. Training in Customer Service Competencies

A face-to-face relationship can contribute to the quality of customized service by enhancing aspects such as customer satisfaction, trust, loyalty, and commitment. Patients tend to be very attuned to the health professional’s “bedside manner” and to such dimensions as empathy, clarity of expression, and listening skills. These attributes, in combination with clinical expertise, can give the patient the sense that his or her own unique situation is being recognized and taken into account as care is being given. Their absence, in fact, may well slow the healing process. While it is certainly the case that face-to-face interaction is resource-intensive and costly, IT-based substitutes have yet to be developed when it is possible. When they are, substantial cost savings will be realized.

In the meantime, some hospitals have developed concierge services designed to meet the unique nonclinical needs of individual patients. Customer-service competencies are required both on the front line and in the back office of any work organization. Nurses represent the front line in the provision of health-care services and, as such, need training that goes beyond the purely clinical. Current initiatives exist, but could be largely reinforced. They can then become advocates for the spread of customization across the organization. Other professionals who are responsible for treatment selection could also benefit from such training.

## The Benefits of Customized Care

How does customized care produce value? Through customization, cost savings can be obtained by targeting relevant actions and suspending or curtailing unnecessary actions, thereby avoiding waste. For example, efforts to prevent food waste in hospitals by using personalized menus have been demonstrated to reduce by 1/3 the quantity of food needed while improving patient satisfaction, with the latter representing a competitive advantage for the hospital.^8^

Using new apps, patients for example with diabetes can also be more directly engaged in improving their dietary habits and physical activity. In general, these apps, tailored to the medical needs of individual patients, can help to improve the health status of both individuals and populations by increasing the probability that patients will follow their prescribed courses of medication and by allowing for the adjustment of medications as indicated.

Another example is in patient transportation. New apps allow patients to get rides to and from their appointments conveniently and on time, helping to maximize efficiency at the treatment site. Customizing transportation increases patient engagement by removing what, for some, is a significant barrier to getting the right care at the right time and place and helps to improve capacity management at the point of service. As a result, long-term cost savings will be realized as the number of missed appointments is reduced and care is thereby improved.

Finally, patients are likely to embrace customization because it is focused on their individual needs. Proof of concept has been developed in other sectors, and some systematic reviews of patient-centered care which involves customization show clinical and economic benefits.^9^ If a pair of shoes or a banking service can be customized, patients might well wonder why customized care and services are not on offer in healthcare. Admittedly, the analogy is somewhere limited but can the healthcare sector really shut its eyes to this mushrooming trend. Some healthcare organizations around the globe have already instituted initiatives of customization which can take different forms, starting with shared decision making, accounting for characteristics needs and preferences of patients, and tailoring interventions. With the development of care customization, different questions need to be solved. For example, additional information that is critical for developing a true profile of patient requires gaining a core sense of an individual’s values and preferences for care. All of this information can be potentially obtained directly from the individual or from inside “big data” repositories. The collected data are probably not the same. Therefore, the process by which these questions are asked and the assessment tools used to obtain this information truly matter.

Even if it raises new challenges, it would truly be a paradox if customization were a feature of all of a patient’s daily activities except those relating to his or her care.

## Ethical issues

Not applicable.

## Competing interests

Author declares that he has no competing interests.

## Author’s contribution

EM is the single author of the paper.

## References

[1] Mannion R, and Exworthy M (2017). (Re) making the procrustean bed? Standardization and customization as competing logics in healthcare”. Int J Health Policy Manag.

[2] Pine JB. Mass Customization: The New Frontier in Business Competition. Boston: Harvard Business Review Press; 1993.

[3] Coulter A, Ellins J (2007). Effectiveness of strategies for informing, educating, and involving patients. BMJ.

[4] Kimberly JR, De Pouvourville G, D’Aunno T. The Globalization of Managerial Innovation in Health Care. Cambridge, UK: Cambridge University Press; 2008.

[5] OECD. Health at a Glance 2015: OECD Indicators. Paris: OECD Publishing; 2015.

[6] Minvielle E, Waelli M, Sicotte C, Kimberly JR (2014). Managing customization in health care: a framework derived from the services sector literature. Health Policy.

[7] Gerves-Pinquie C, Daumas-Yatim F, Lalloue B (2017). Impacts of a navigation program based on health information technology for patients receiving oral anticancer therapy: the CAPRI randomized controlled trial. BMC Health Serv Res.

[8] Züger G, Honegger F (2014). Essential Requirements for the Parameterization of Food Waste in Hospitals. International Journal of Facility Management.

[9] Rathert C, Wyrwich MD, Boren SA (2013). Patient-centered care and outcomes: a systematic review of the literature. Med Care Res Rev.

